# The predictive power of baseline metabolic and volumetric [^18^F]FDG PET parameters with different thresholds for early therapy failure and mortality risk in DLBCL patients undergoing CAR-T-cell therapy

**DOI:** 10.1016/j.ejro.2024.100619

**Published:** 2024-12-17

**Authors:** Emil Novruzov, Helena A. Peters, Kai Jannusch, Guido Kobbe, Sascha Dietrich, Johannes C. Fischer, Jutta Rox, Gerald Antoch, Frederik L. Giesel, Christina Antke, Ben-Niklas Baermann, Eduards Mamlins

**Affiliations:** a Department of Nuclear Medicine, Medical Faculty and University Hospital Duesseldorf, Heinrich Heine University Duesseldorf, Düsseldorf 40225, Germany; b Department of Diagnostic and Interventional Radiology, Medical Faculty and University Hospital Duesseldorf, Heinrich-Heine-University Duesseldorf, Düsseldorf 40225, Germany; c Center for Integrated Oncology Aachen Bonn Cologne Düsseldorf (CIO ABCD), Germany; d Department of Hematology, Oncology and Clinical Immunology, Medical Faculty and University Hospital Düsseldorf, Heinrich-Heine-University Düsseldorf, Düsseldorf 40225, Germany; e Institute for Transplantation Diagnostics and Cellular Therapy, University Hospital Düsseldorf, Düsseldorf, Germany; f Institute for Radiation Sciences, 2-2 Yamadaoka, Suita, Osaka 565-0871, Japan

**Keywords:** CAR-T-cell therapy, FDG PET, Metabolic tumor burden, Volumetric PET parameter

## Abstract

**Objective:**

[^18^F]FDG imaging is an integral part of patient management in CAR-T-cell therapy for recurrent or therapy-refractory DLBCL. The calculation methods of predictive power of specific imaging parameters still remains elusive. With this retrospective study, we sought to evaluate the predictive power of the baseline metabolic parameters and tumor burden calculated with automated segmentation via different thresholding methods for early therapy failure and mortality risk in DLBCL patients.

**Materials and methods:**

Eighteen adult patients were enrolled, who underwent CAR-T-cell therapy accompanied by at least one pretherapeutic and two posttherapeutic [^18^F]FDG PET scans within 30 and 90 days between December 2018 and October 2023. We performed single-click automatic segmentation within VOIs in addition to extracting the SUV parameters to calculate the MTVs and TLGs by applying thresholds based on the concepts of a fixed absolute threshold with an SUV_max_ > 4.0, a relative absolute threshold with an isocontour of > 40 % of the SUV_max_, a background threshold involving the addition of the liver SUV value and its 2 SD values, and only the liver SUV value.

**Results:**

For early therapy failure, baseline metabolic parameters such as the SUV_max_, SUV_peak_ and SUV_mean_ tended to have greater predictive power than did the baseline metabolic burden. However, the baseline metabolic burden was superior in the prediction of mortality risk regardless of the thresholding method used.

**Conclusion:**

This study revealed that automated delineation methods of metabolic tumor burden using different thresholds do not differ in outcome substantially. Therefore, the current clinical standard with a fixed absolute threshold value of SUV > 4.0 seems to be a feasible option.

## Highlights

• The predictive power of baseline metabolic parameters for early therapy failure outperforms that of volumetric parameters.
• Given the mortality risk, baseline volumetric parameters had a greater predictive value than metabolic parameters though.
• The deployment of the fixed absolute threshold value of SUV > 4.0 seems to be a feasible option compared with other methods.

## Graphical Abstract

**Figure:**
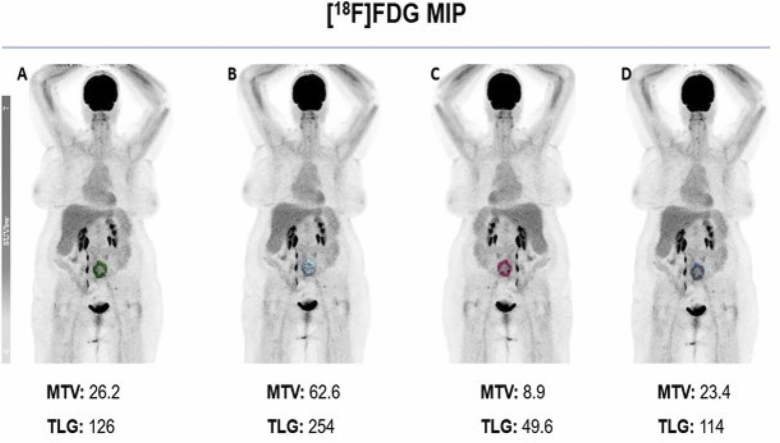

## 1 Introduction

In the last two decades, immunochemotherapy has been a mainstay of patient management for patients with hematological malignancies. Despite great initial success with the R-CHOP protocol (rituximab in combination with cyclophosphamide, hydroxydaunorubicin, vincristine, and prednisone) as a part of first-line treatment, up to 30 % of patients with aggressive B-cell lymphoma experience disease progression or early relapse. This phenomenon appears to be nonresponsive or refractory to even further second- or third-line treatments, such as chemotherapy or autologous stem cell transplantation, and is associated with poor outcomes [1]. Hence, further research on a more effective targeting strategy accompanied by an immune response has paved the way for the development of various chimeric antigen receptor (CAR) T-cell therapies, which can be depicted as a sophisticated form of engineered cellular immunotherapy. The pan-B-cell marker CD19 represents the most common target antigen and can be selectively recognized by the CAR. The core of this approach is the viral transduction of the genetic plan for a CAR and its costimulatory domains into patient-derived T cells. A single infusion of replicative CAR-T cells and their persistence enable long-term disease control without further treatment. Hence, this therapy is also regarded as a living drug because of the long-term persistence of engineered cells in the body. Since CAR-T-cell infusion begins to have effects mostly in the first two weeks, adverse effects such as cytokine-release syndrome (CRS) or immune effector cell-associated neurotoxicity syndrome (ICAN) are mostly observed during this time interval [2], [3], [4]. The approach with CAR-T-cell therapy delivered impressive clinical results, with a durable complete remission rate of up to 40–50 % for therapy-refractory or multiple relapsed DLBCLs in the first three years of follow-up [5].

In addition to various cancer immunotherapies, therapy management with this approach requires even more intensive utilization of [^18^F]FDG PET/CT, which involves both pretherapeutic and posttherapeutic periods, because of its excellent accuracy in the detection of lymphoproliferative processes in B-cell lymphoma and determination of immunotherapy-related side- and adverse effects [4]. [^18^F]FDG scanning has been recommended at the time of decision (TD), time of treatment or just prior to CAR-T-cell infusion after the completion of bridging therapy and lymphodepletion; early and late follow-up at 30 (T1) and 90 (T2) days; and long-term monitoring at 1 year in the posttherapeutic period [3]. However, the current clinical practice seems to have reached a consensus by conducting one pretherapeutic scan and subsequent follow-ups at T1 and T2 via [^18^F]FDG. This specific timing of posttherapeutic scans is supposed to determine in a timely manner which patients show a response (decrease in lesion size and/or [^18^F]FDG uptake) or nonresponse (lack of complete metabolic response) within the first 30 days and treatment failure, whereas treatment failure is classified as early or late accordingly if it is observed within or after the first 90 days after treatment [4], [6]. Reporting systems in clinical practice are mainly based on the Lugano classification, particularly the Deauville scoring (DS) system with a 5-point scale, which is a visual-qualitative, nonimmunotherapy-specific response criterion with limited predictive value for patient outcome or stratification for proper therapy management [7].

Therefore, semiquantitative metabolic parameters, such as various derivatives of standard uptake volume (SUV), and volumetric analyses, such as metabolic tumor volume (MTV) and total lesion glycolysis (TLG) at baseline, as well as follow-up scans, have been deployed to investigate their predictive value for therapy outcomes to enable more proper patient selection for CAR-T-cell therapy and optimize the clinical decision-making process for the initiation of other tumor burden-reducing treatment protocols [4].

Recent data underscore the role of baseline PET parameters in the prediction of prognosis and adverse effects of CAR-T-cell therapy [3]. The MTV and TLG calculated via user- or algorithm-defined segmentation on the basis of threshold values or algorithms within a manually or semiautomatically drawn region of interest (ROI) have been shown to reflect the metabolic burden better than SUV parameters. These volumetric parameters have been proven to be calculated more precisely and reliably and are practically more applicable with the application of certain threshold values, such as a fixed, relative or background value. The fixed absolute threshold is set in this context by an SUV value of 2.0–5.0, whereas the fixed relative threshold is set by a cutoff percentage of the SUV_max_ of the lesion of 40–60 %. In contrast, volumetric analysis by background thresholding involves adding a liver SUV value and its 1 or 2 standard deviations (SDs), which may be more precise and patient- and scan-specific but also a time-consuming method [8]. Thus, the precise contributions of different methodical approaches in the calculation of volumetric PET parameters and their relationships with metabolic PET parameters during CAR-T-cell therapy management remain elusive.

The aim of this study was to investigate the predictive power of metabolic and volumetric PET parameters with different thresholds with respect to relevant clinical outcomes to determine the specific prognostic impact of these PET parameters in terms of therapy responsiveness, early therapy failure and mortality risk in a comparative manner.

## 2 Materials and methods

### 2.1 Study design and population

We retrospectively analyzed 18 adult patients with relapsed or refractory diffuse large-cell B-cell lymphoma (r/r-DLBCL) who underwent CAR-T-cell therapy accompanied by at least one pretherapeutic and two posttherapeutic [^18^F]FDG PET scans within 30 and 90 days after CAR-T-cell infusion, respectively, according to our institutional guidelines between December 2018 and October 2023. Patients were followed up by querying medical records and clinical data. Assessment of the primary lesions, extranodal lesions, and distant metastases was confirmed from the results of biopsy, surgery, imaging data, or long-term patient follow-up. Table 1 shows the baseline characteristics of the enrolled patients. The data were anonymized and retrospectively analyzed. The study was reviewed and approved by the Ethical Committee of the Medical Faculty of Heinrich Heine University Duesseldorf, Germany (study number: 2023--2618) and was conducted in accordance with the national and international guidelines as well as the Declaration of Helsinki.

**Table 1:** Patient characteristics prior to CAR-T-cell infusion.

Total number of patients	18
Gender (male vs. female)	10 vs. 8
Age (mean ± SD)	60 ± 12
Therapy lines prior to CAR T-cell infusion	
Chemotherapy	18 (100 %)
Immunotherapy	17 (94 %)
Radiotherapy	3 (17 %)
Stem cell transplantation	5 (28 %)
Bridging Therapy	18 (100 %)
Infused CAR T-cell product	
Axicabtagen-Ciloleucel (Yescarta®)	10
Tisagenleucel (Kymriah®)	8
Time Interval between baseline [^18^F]FDG scan and CAR T-cell Infusion in days (mean ± SD)	15 ± 17
Time Interval between CAR T-cell Infusion and T1 [^18^F]FDG scan in days (mean ± SD)	32 ± 7
Time Interval between CAR T-cell Infusion and T2 [^18^F]FDG scan in days (median; range)	118 ± 44
Injected activity [^18^F]FDG in MBq (mean ± SD)	231 ± 38
PET scan acquisition time in minutes (mean ± SD)	63 ± 7
Follow-up period after CAR T-cell Infusion in months (median; range)	11 (6 – 45)
Progression-free-survival (PFS) in months (median; range)	3 (1−27)

### 2.2 [^18^F]FDG PET/CT acquisition

The whole-body and total-body enhanced or nonenhanced PET/CT scans were performed approximately 60 minutes after intravenous injection of body weight-adapted (3 MBq/kg) [^18^F]FDG, with a mean of 231 ( ± 38) MBq on the hybrid PET/CT scanner (Siemens Biograph 128 mCT) that possesses EANM Research Ltd. (EARL) accreditation, as indicated by the European Association of Nuclear Medicine (EANM). Supplemental Table S1 summarizes the scan protocol.

### 2.3 Imaging analysis

All [^18^F]FDG PET/CT scans were assessed by two nuclear physicians (EN and EM) and two radiologists (HP and KJ). PET images were originally assessed visually according to the Deauville score (DS) and classified as complete metabolic response (CMR) with DS 1–3, partial metabolic response (PMR) with DS 4–5, or progressive disease (PD). Image analysis was performed via a dedicated software package (Hermes, Affinity 3.0.5; Hermes Medical Solutions, Stockholm, Sweden). The lymphoma manifestations were assessed by a series of manually drawn constraint ROIs, of which corresponding VOIs were created by the software. The VOIs were then manually adjusted to ensure that any spillover effects from neighboring organs or structures exhibiting [^18^F]FDG uptake greater than background were excluded, as this could introduce bias into our results. Reference regions were manually drawn as spherical VOIs in the thoracic aorta and liver.

We performed single-click automatic segmentation within VOIs in addition to extracting the SUV parameters to calculate the MTVs and TLGs (MTV × SUV_mean_ (obtained from the corresponding MTV)) by applying thresholds, which we determined on the basis of the concepts of a fixed absolute threshold with an SUV_max_ value of > 4.0, a relative absolute threshold with > 40 % of the SUV_max_, and a background threshold involving adding a liver SUV value with 2 SD values. In addition to these verified methods, we assessed the liver SUV value as a further thresholding tool within the aforementioned dedicated software platform. Additionally, given the recent data regarding the clinical significance of intratumoral heterogeneity in DLBCL, we analyzed its effect by assessing the metabolic heterogeneity index (HI), which is determined by the quotient of the SUV_max_ and the SUV_mean_ of the lesion [9], [10].

### 2.4 Statistical analysis

Clinical and demographic characteristics are presented via descriptive statistics. Comparative analyses of semiquantitative parameters and metabolic burden (MTV and TLG) were performed via t tests and nonparametric Mann–Whitney U tests. A p value of < 0.05 was considered statistically significant. The descriptive statistical analyses were performed via Excel Version 2311 (Microsoft® Excel® 2021 MSO) and SigmaPlot 11.0 (Systat Software Inc.). Receiver operating characteristic (ROC) curve analysis and the area under the ROC curve (AUC) were used to compare the predictive power of the clinical and imaging parameters for patient outcome. To this end, the sensitivity, specificity, optimal cutoff value, and 95 % confidence interval (CI) were calculated for each parameter via MedCalc Software (MedCalc® Statistical Software version 20.011). Optimal cutoffs were defined by Youden’s index as those resulting in high sensitivity corresponding to the highest negative predictive value or the maximum specificity for a given minimum level of sensitivity. The comparison of different AUCs was conducted via the method described by DeLong et al. [11].

We used logistic regression to determine the associations between the assessed parameters and the event of interest, and Kaplan—Meier survival curves and univariate Cox regression tests were used to examine the correlations between the predictors under investigation. Hazard and odds ratios, including 95 % confidence intervals (CIs), are reported. Surviving patients were censored at the last follow-up, and only death was considered an event. Progression-free survival (PFS) was defined as the time from the infusion of CAR-T cells to relapse, disease progression, death from any cause or the follow-up cutoff date, whereas overall survival (OS) was defined as the time in months from CAR-T-cell infusion to either death by any cause or the follow-up cutoff date. In this study, data cleaning was performed to identify and correct any errors, inconsistencies, or missing values in the dataset, thus improving the overall quality and reliability of the data.

## 3 Results

### 3.1 Clinical and baseline PET characteristics

Table 2 shows the clinical and imaging parameters of the baseline PET scan in the pretherapeutic period, which were evaluated to determine their prognostic impact on patient outcome. The baseline [^18^F]FDG scans revealed overall intensive metabolic activity in terms of high SUV_max_ values and correspondingly moderate to high metabolic tumor burdens, in line with an overall Deauville score of 4 or 5, with the exception of only three patients. Approximately 83 % of patients suffer from cytokine release syndrome (CRS), with only 1 patient from Grade 3 CRS; all of these patients were treated successfully according to institutional guidelines, whereas only 3 patients displayed self-limiting, temporary ICANS symptoms.

**Table 2:** Overview of the clinical and PET imaging parameters of the baseline scan.

**Clinical Characteristics**	
LDH level at CAR T-cell infusion (median; range)	218 (107 – 798)
CRP level at CAR T-cell infusion (median; range)	0.75 (0.1 – 11.8)
WBC level at CAR T-cell infusion (median; range)	6400 (1400–32000)
IL−6 level at CAR T-cell infusion (median; range)	26.7 (3 – 45127)
Ki−67 index (median; range)	80 (29 – 90)
**CRS Grade**	
Grade 0 Grade I Grade II Grade III	3 (17 %) 11 (61 %) 3 (17 %) 1 (5 %)
**ICAN Grade**	
Grade 0 Grade I Grade II Grade III Grade IV	15 (85 %) 0 (0 %) 1 (5 %) 1 (5 %) 1 (5 %)
**Baseline [**^**18**^**F]FDG PET Characteristics**	
SUV_max_ (median; range)	8.9 (1.8 – 39.5)
SUV_peak_ (median; range)	6.4 (1.3 – 35.8)
SUV_mean_ (median; range)	2.9 (1.2 – 10.7)
*Fixed absolute threshold with SUV > 4,0*	
Total MTV (median; range)	26.2 (0.1 – 2242)
Total TLG (median; range)	126 (0.1 – 17214)
*Fixed Relative threshold with 40 % Isocontour*	
Total MTV (median; range)	32.2 (0.1–491)
Total TLG (median; range)	254 (0.1–6319)
*Background threshold with Liver SUV + 2 SD*	
Total MTV (median; range)	8.9 (0.1–5088)
Total TLG (median; range)	49.6 (0.1–24542)
*Background threshold with only Liver SUV*_*max*_	
Total MTV (median; range)	23.4 (0.1 – 5782)
Total TLG (median; range)	114 (0.1–25649)

We evaluated our patient cohort with respect to therapy response at T1 and T2 after CAR-T-cell infusion to detect therapy responsiveness and early failure, respectively, as well as mortality during further follow-up. The diagnosis of disease progression was confirmed from the results of biopsy, clinical findings and imaging data other than PET data. The corresponding subgroups were investigated via the clinical and imaging parameters depicted in Table 1 *&* 2.

### 3.2 Prediction of therapy responsiveness and early failure

**Fig. 1:**
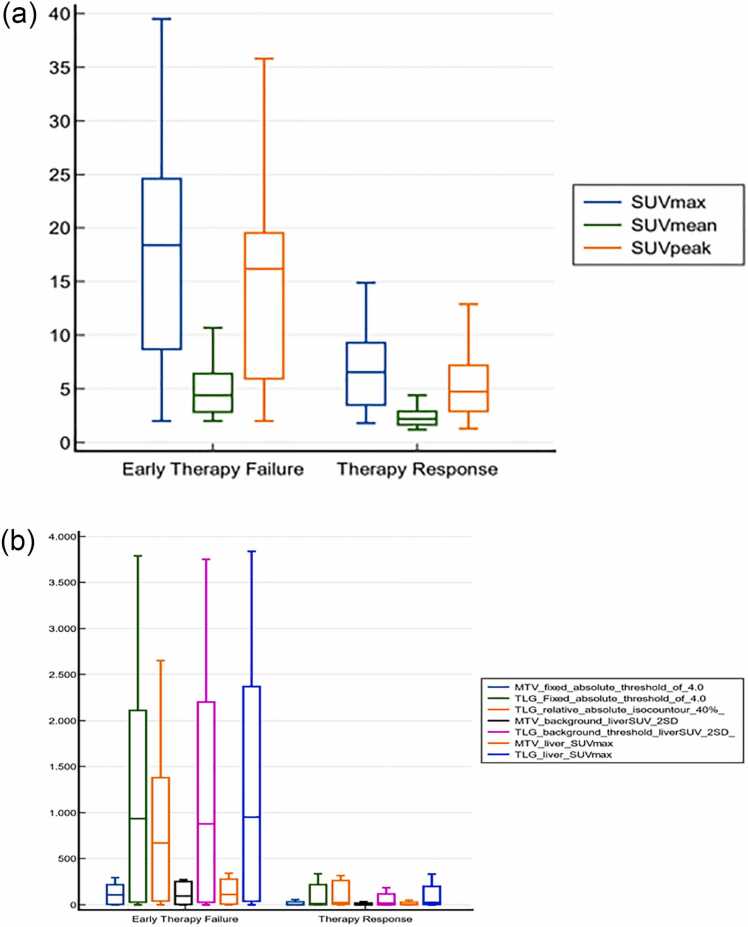
Graphical illustration of metabolic and volumetric PET parameters at baseline with respect to early therapy response **a)** metabolic parameters **b)** volumetric parameters.

The intergroup analysis with respect to therapy responsiveness did not yield a statistically significant result for the predictive value of any clinical, laboratory or imaging parameter. In contrast, the intergroup assessment of a variety of parameters, depicted in Fig. 1 *&* Table 3, concerning early therapy failure at T1 after CAR-T-cell infusion yielded statistically significant results. While all of the metabolic and volumetric PET parameters, with the exception of the MTV, which is based on a fixed relative threshold value, yielded significant results, C-reactive protein (CRP) at CAR-T-cell infusion appeared to be the only statistically significant nonimaging predictive parameter. We subsequently performed receiver operating characteristic (ROC) analysis on these statistically significant variables to determine cutoff values for establishing discriminatory thresholds for early therapy failure.

**Table 3:** Overview of the results of ROC, logistic regression and Cox regression analyses (univariate analysis) for laboratory findings and metabolic and volumetric PET parameters (p < 0,05) in patients with early therapy failure.

	Cutoff Value	Sensitivity	Specificity	AUC (95 % CI)	Odds Ratio *P value*	Hazards Ratio 95 % (CI) *P value*
CRP	1.5	55.5	100	0.802	2.76 *0.15*	26.5 2.96 – 237.9 *0.003*
SUV_max_	14.9	66.6	100	0.854	1.22 *0.04*	9.42 2.23 – 39.76 *0.002*
SUV_peak_	12.9	66.6	100	0.833	1,24 *0.04*	9.42 2.23 – 39.76 *0.002*
SUV_mean_	3.1	66.6	87.5	0.854	2.87 *0.054*	3.27 1.12 – 9.59 *0.03*
MTV fixed absolute	56.6	66.6	100	0.792	1.02 *0.12*	9.42 2.23 – 39.76 *0.002*
TLG fixed absolute	337	66.6	100	0.806	1.0 *0.13*	3.88 1.27 – 11.89 *0,01*
TLG relative absolute	317	66.6	100	0.792	1.0 *0.18*	9.42 2.23 – 39.76 *0.002*
MTV background	34	66.6	100	0.792	1.03 *0.16*	9.42 2.23 – 39.76 *0.002*
TLG background	327	66.6	100	0.806	1.0 *0.15*	9.42 2.23 – 39.76 *0.002*
MTV liver	50,6	66.6	100	0.806	1.02 *0.16*	9.42 2.23 – 39.76 *0.002*
TLG liver	333	66.6	100	0.806	1.0 *0.15*	9.42 2.23 – 39.76 *0.002*

**Fig. 2:**
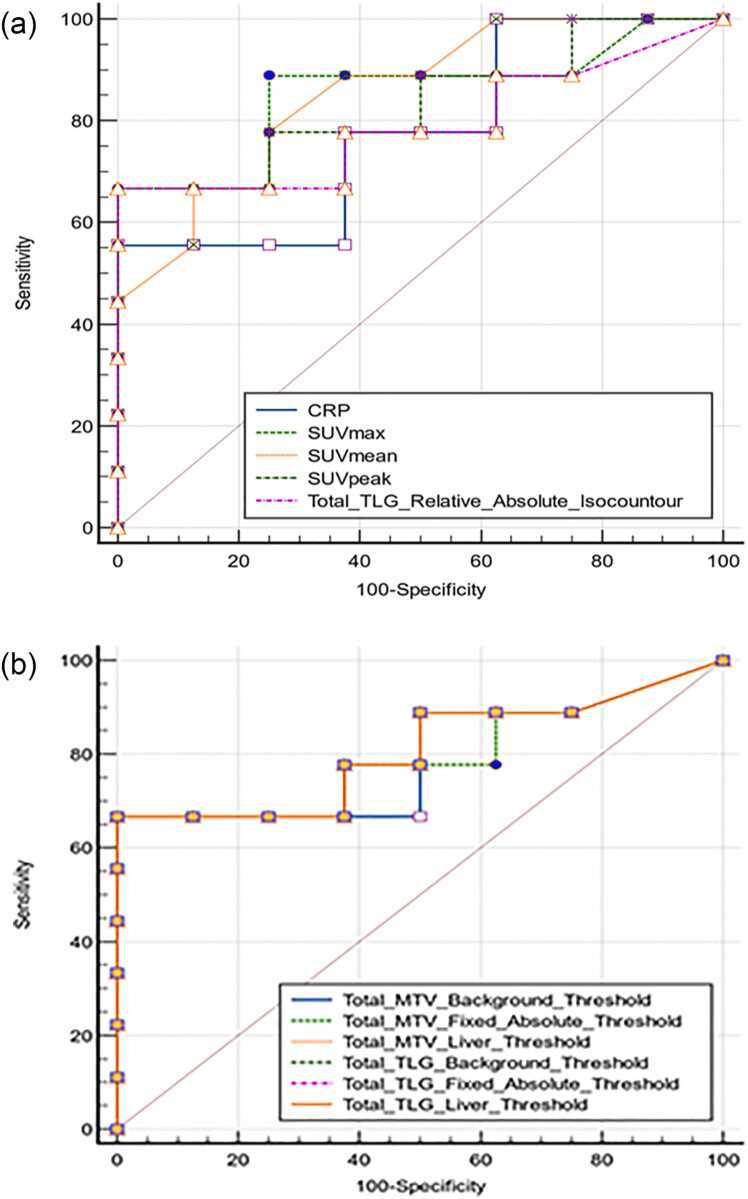
An overview of the comparison of ROC curves of metabolic (**a**) and volumetric (**b**) PET parameters for the cohort with early therapy failure.

In particular, the discriminatory power of metabolic PET parameters seemed to be better than that of volumetric PET parameters, whereas the comparison of ROC curves revealed no statistically significant difference (Fig. 2 & Table 3). Thus, given the relatively small cohort, we proceeded with univariate logistic regression to assess the specific strength of the associations of the variables with the outcome, whereas multivariate analysis was not pursued for further analysis because of inherent collinearity of the assessed PET parameters. The SUV_max_ and SUV_peak_ parameters were the only statistically significant results, with similar odds ratios of 1.22 and 1.24 (p values: 0.04), respectively. In essence, metabolic PET parameters, in terms of the SUV_max_ and SUV_peak,_ seemed to be more strongly associated with early therapy failure than total tumor burden was in terms of volumetric PET parameters.

Furthermore, we assessed the associations between the abovementioned parameters and early therapy failure via survival analysis via Cox regression analysis with a hazard ratio (HR), which allows us to statistically consider the time-to-event nature of the outcome while accommodating censoring and varying follow-up times. Both metabolic and volumetric parameters displayed statistically significant hazard ratios at nearly the same level, indicating an increased risk of disease progression, as most of the PET parameters revealed an HR of 9.42 (95 % CI: 2.23–39.76, p = 0.002), probably due to either a small cohort size or collinearity of these parameters. Thus, the impacts of both the intergroup analysis of metabolic and volumetric parameters and the intragroup analysis of volumetric parameters based on various calculation methods appeared to be very similar when patient outcomes were considered. Notably, we performed logistic and Cox regression analyses according to the cutoff values of the ROC analysis. Without the cutoff value, the SUV_max_ was the only statistically significant HR (1.04; 95 % CI: 1.00–1.09) of the parameter, whereas logistic regression analysis revealed a significant output only with respect to the threshold cutoff values.

### 3.3 Prediction of mortality risk

**Fig. 3:**
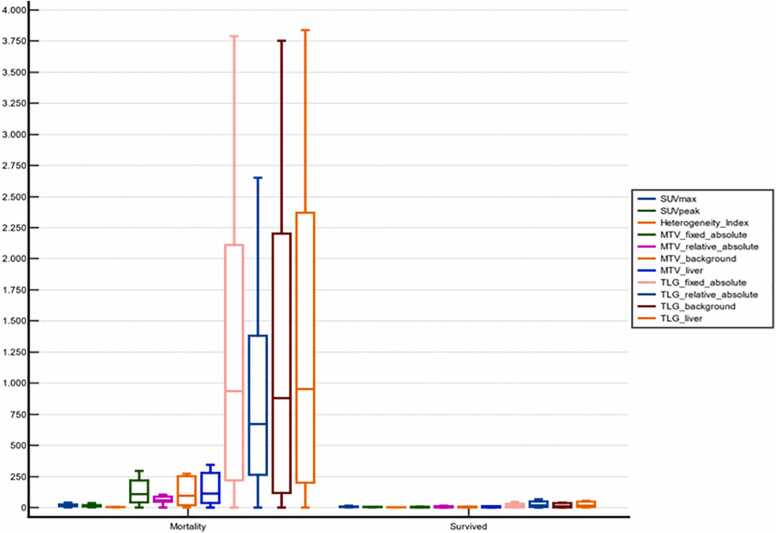
Graphical illustration of metabolic and volumetric PET parameters at baseline with respect to mortality.

**Fig. 4:**
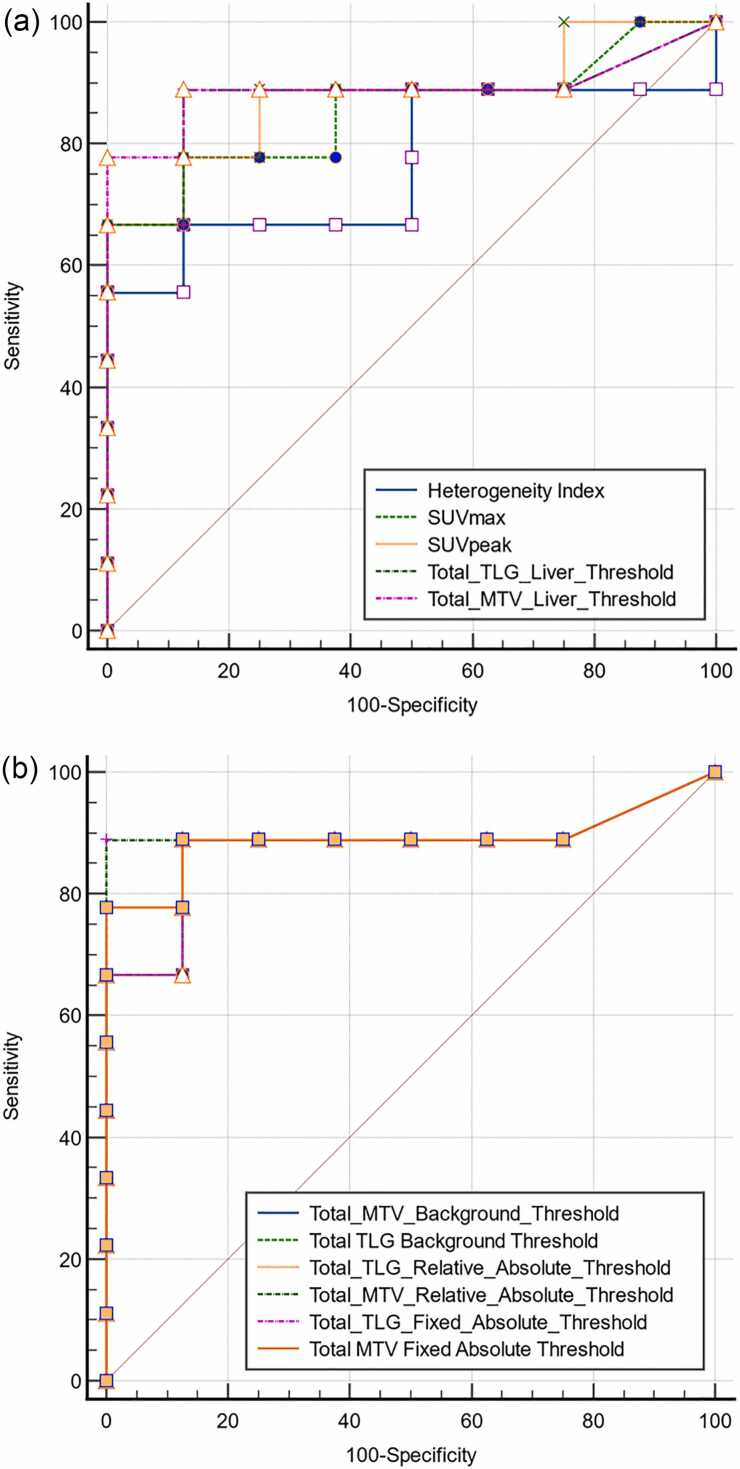
Overview of the comparison of ROC curves of metabolic and volumetric PET parameters with respect to mortality risk (a, b).

We investigated the value of clinical and positron emission tomography (PET) parameters in predicting mortality. Essentially, the patients who died during the clinical course presented numerically higher values of metabolic and volumetric parameters (Fig. 3). In addition to the volumetric PET parameters used to assess the tumor burden at baseline, only the SUV_max,_ SUV_peak_ and HI from the metabolic PET parameters were significantly different between the cohorts. We subsequently performed ROC analysis on these statistically significant variables to determine cutoff values for establishing predictive thresholds of mortality. The volumetric PET parameters, indicators of tumor burden, tended to better predict the outcome than did the metabolic PET parameters and HI, while the statistical comparison of the ROC curves revealed no significant difference (Fig. 4). Further analysis with univariate logistic regression revealed significant results only for SUV_max_ and SUV_peak,_ with odds ratios of 1,24 and 1,31 (p values: 0,04 and 0,03), respectively (Table 4). Owing to collinearity of the assessed variables and the small cohort size, a multivariate analysis was considered to be unsuitable.

**Table 4:** Overview of the results of the ROC, logistic regression and Cox regression analyses (univariate analysis) for metabolic and volumetric PET parameters (p < 0,05) for mortality risk.

	Cutoff Value	Sensitivity	Specificity	AUC (95 % CI)	Odds Ratio *P value*	Hazards Ratio 95 % (CI) *P value*
SUV_max_	14.9	66.6	100	0.854	1.24 *0.04*	1.05 0 – 0.11 *0.06*
SUV_peak_	12.9	66.6	100	0.875	1.31 *0.03*	1.06 0 – 0.11 *0.07*
MTV fixed absolute	36.3	77.7	100	0.889	1.07 *0.08*	1.0 0 – 1.0 *0.04*
TLG fixed absolute	45.5	88.8	87.5	0.875	1.01 *0.14*	1.0 0 – 1.0 *0.03*
MTV relative absolute	32.2	88.8	100	0.903	1.09 0.02	1.01 0 – 1.01 *0.02*
TLG relative absolute	65.9	88.8	87.5	0.875	1.01 *0.06*	1.0 0 – 1.0 *0.02*
MTV background	7.9	88.8	87.5	0.875	1.06 *0.16*	1.0 0 – 1.0 *0.06*
TLG background	39.7	88.8	100	0.875	1.0 *0.17*	1.0 0 – 1.0 *0.04*
MTV liver	35.5	77.7	100	0.889	1.07 *0.11*	1.0 0 – 1.0 *0.06*
TLG liver	55.1	88.8	100	0.875	1,01 *0.17*	1.0 0 – 1.0 *0.04*
Heterogeneity Index	3.38	55.5	100	0.764	2,7 0.07	2.09 1.37 – 3.93 *0.02*

Cox regression analysis with HRs revealed statistically significant results only for the volumetric PET parameters and HI. Thus, the various methods used to calculate the tumor burden seemed to have the same predictive power for mortality risk, while the HI displayed a higher hazard ratio (HR) of 2,09 (95 % CI: 1,37–3,93) than did the volumetric parameters. Eventually, the tumor burden at the ^18^F[FDG] baseline scan seems to represent a substantial predictive factor for mortality risk after CAR-T-cell therapy in DLBCL patients.

## 4 Discussion

CAR-T-cell therapy is one of the most effective, novel cancer immunotherapies and appears to be effective, especially in highly aggressive, relapsed or therapy-refractory hematological malignancies such as DLBCL. This therapy presents some challenges and drawbacks, including high costs and immunomodulation-specific adverse effects such as CRS, ICANS, and hematotoxicity. Despite the fact that this is associated with an overall long-term success rate of up to 50 %, even patients receiving CAR-T-cell therapy with a limited response have been shown to experience therapeutic benefit in terms of prolonged overall survival due to newly induced favorable responsiveness to conventional treatment modalities. Given the multifaceted nature of CAR-T-cell therapy, [^18^F]FDG scanning, as a consensus in clinical practice, has been incorporated into every step of this therapeutic process, first as a baseline scan just prior to CAR-T-cell reinfusion and then as an early follow-up control tool within 30 and 90 days of the therapeutic period. This approach is supposed to enable effective and target-oriented patient preselection as well as timely therapy management changes in cases of therapy nonresponsiveness or early therapy failure, as PFS and OS are still the scope of ongoing research in this field for the determination of reliable, predictive factors. However, the existing data in the literature are inconsistent regarding the segmentation methods used to determine metabolic tumor burden and, in particular, the specific associations of different imaging parameters with outcomes [2], [3], [4].

With our single-center, retrospective study, we aimed to contribute to the growing body of evidence in the current literature by, to the best of our knowledge, for the first time, critically evaluating automated segmentation methods of volumetric PET parameters with different thresholding and metabolic parameters as well as the metabolic intratumoral heterogeneity comprehensively within the same cohort and their prognostic impact on patient outcome in terms of early therapy failure (within 90 days after CAR-T-cell reinfusion) and mortality risk. In this context, a consistent, robust, user-friendly thresholding method for assessing metabolic tumor burden is still lacking. In contrast to previous studies, we focused specifically on evaluating the risk of early disease progression and mortality among those who experienced the event, as censored cases in conventional PFS and OS analysis included individuals who had not experienced the event of interest by the end of the study period.

Our study has some limitations such as its retrospective nature and relatively limited cohort size. These might introduce potential biases, including patient selection and data collection. In addition, the small cohort size of 18 patients might influence the power of statistical analysis and limit the generalizability of our conclusions. Finally, our method for calculating and segmenting metabolic burden relies on dedicated software, which might be accessible only in large, high-end centers.

### 4.1 Predictive value of imaging parameters for early therapy failure

Despite comprehensive volumetric imaging analysis with automated segmentation with different thresholding alongside metabolic and clinical parameters, we could not define any parameter with substantial predictive power for therapy nonresponsiveness within 30 days following CAR-T-cell reinfusion, which is in line with the results of Vercellino et al. [12]. Further analysis regarding early therapy failure revealed significant results for all metabolic and volumetric parameters, with the exception of the MTV, which is based on the relative absolute threshold and is likely due to heterogeneous tracer uptake. Notably, the discriminatory power of the metabolic parameters with SUV_max_, SUV_mean_ and SUV_peak_ tended to be greater than that of the volumetric parameters; however, there was no significant result in a comparative analysis of the ROC curves (Fig. 2 & Table 3). Univariate logistic regression with cutoff values determined by receiver operating characteristic (ROC) analysis revealed substantial associations between the SUV_max_ and SUV_peak_ parameters at cutoff values of 14.9 and 12.9, respectively, and early therapy failure. Nevertheless, considering the constant hazard ratio of 9.2 after Cox regression analysis for all the imaging parameters despite different automated segmentation methods of baseline tumor burden, the impact of these parameters appears to have an equivalent predictive power for early therapy failure, even though SUV values have emerged as more practical methods in clinical practice. In addition, there was no substantial difference among segmentation methods with different thresholds in predicting early therapy failure, where all MTV and TLG cutoff values ranged from 34.0–56.6 and 317.0–333.0, respectively.

In addition to the imaging parameters, CRP was the only nonimaging parameter that was demonstrated to be associated with the risk of early therapy failure, concordant with the conclusion of Vercellino et al. In addition, they demonstrated that an MTV > 80 had relevant discriminatory power on the basis of segmentation with a threshold of an SUV > 4.0, whereas our cohort revealed a cutoff value of 56.6 on the basis of the same threshold [12]. A retrospective study with 22 patients investigating the predictive power of baseline FDG scans conducted by Georgi et al. revealed a relevant discriminatory power at a value of > 25 ml of MTV, which was calculated on the basis of segmentation with a threshold of an SUV of 3.0. Despite the even lower threshold SUV value than that in our study, the MTV was found to be lower than that in our study, presumably owing to prior bridging therapy. Notably, the subgroup with therapy failure had a median SUV_max_ of 21.2 [13]. The cohort of Cohen et al. included two baseline PET scans at the time of decision (TD) and time of transfusion (TT), of which the TT results seem to be more closely related to our findings. This subgroup displayed a significant effect on therapy failure when the TT-SUV_max was_ > 12.1, whereas the cutoff value in our cohort was similar to that when the SUV_max_ was > 12.9 [14].

Interestingly, the study of Wang et al. demonstrated no significant predictive power of baseline metabolic burden despite the relatively high MTV and TLG values of the subgroup with progression. However, they could display the discriminatory power of the baseline metabolic burden for the prediction of immunotherapy-related adverse effects, such as CRS or ICANS. Therefore, this was probably a biased effect due to the small cohort size [15], [16], [17]. In our cohort, however, patients experienced a low-grade CRS episode with only one severe case (grade 3 CRS), while we observed only two high-risk ICANS episodes; thus, we refrained from further evaluation. Bridging therapy might be the leading cause for the low incidence of high-risk immunotherapy-related adverse effects as well as the relatively lower baseline metabolic tumor burden in our cohort. Furthermore, the metabolic intratumoral heterogeneity index (HI) did not substantially differ for the prediction of early disease progression.

Taken together, our study demonstrated for the first time that not only the SUV_max_ but also all metabolic PET parameters display substantial predictive power for early therapy failure, where the SUV_peak_ and SUV_max_ are equivalent to the baseline tumor burden. Given the strong agreement in predictive power among the metabolic burden delineation methods, the current clinical standard with a fixed absolute threshold value of SUV > 4.0 appears to be an appropriate cutoff value for the determination of MTV and TLG [18], [19].

### 4.2 Predictive value of imaging parameters for mortality risk

Analysis of clinical parameters, i.e., LDH or CRP levels at retransfusion, and imaging parameters related to mortality risk revealed a relevant predictive value only for imaging parameters. Overall, the baseline imaging parameters were greater in patients who died during follow-up (Fig. 3). Among the metabolic PET parameters, only the SUV_max_ and SUV_peak_ were found to display a relevant discriminatory power, with areas under the ROC curves of 0.854 and 0.875, respectively, at > 14.9 and > 12.9, respectively. However, Cox regression analysis revealed that the hazard ratios for these parameters were not associated with the risk of death. In contrast, all the parameters depicting automated segmentation-based metabolic tumor burden with different thresholding methods revealed relevant discriminatory power, with an area under the ROC curve > 0.875 (range of > 0.875–0.903). A comparative analysis of the ROC curves, however, revealed no significant results for this subgroup (Fig. 4). In addition, these parameters exhibited statistically significant Cox regression-calculated hazard ratios, with the exception of the calculation of MTV on the basis of background thresholding, although the association did not appear to be statistically strong (Table 4).

**Fig. 5:**
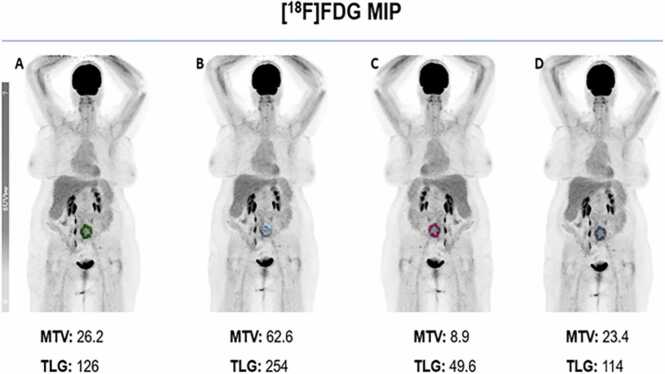
Exemplary automated segmentation of the baseline tumor burden with 4 different thresholding methods in a 67-year-old female patient with abdominally relapsed DLBCL: **A)** fixed absolute threshold of SUV_max_ > 4.0, **B)** fixed relative threshold of > 40 % **C)** background threshold with a liver SUV value + 2 SDs, **D)** background threshold with a liver SUV value.

The aforementioned analyses imply a better predictive power of the baseline metabolic burden for the risk of death than do metabolic PET parameters. Moreover, there was only a slight difference among the delineation methods with different thresholds, although background thresholding (with only an SUV value of liver and liverSUV+2 SD) did not seem as effective as the other competing methods did (Fig. 5). Another interesting result in this subgroup was that metabolic intratumoral heterogeneity, to the best of our knowledge for the first time in a DLBCL cohort receiving CAR-T-cell therapy, was significantly associated with mortality risk, with an HR of 2.09 (CI: 1.37–3.93). Our results are in concordance with the initial evidence in the literature indicating the role of intratumoral heterogeneity in the effect of DLBCL on patient outcome [10].

Since the literature data have focused solely on the analysis of overall survival as an endpoint of studies, a direct comparative evaluation with our results does not seem to be suitable. Nevertheless, data from the literature suggest an association between a lower baseline metabolic tumor burden and survival and response rates [20], [21], [22], [23]. There is, however, a discrepancy regarding the specific association of MTV or TLG with the survival rate, as Ababneh et al. highlighted the role of TLG in the survival rate while underscoring the impact of the MTV on disease progression [24].

## 5 Conclusion

In conclusion, our study demonstrated that the metabolic parameters SUV_max_ and SUV_peak_ have equivalent predictive power but outperform the baseline metabolic tumor burden for early therapy failure. In contrast, baseline metabolic tumor burden, in terms of both MTV and TLG, appeared to have greater predictive value than metabolic parameters for mortality risk. Furthermore, we showed that automated delineation methods of metabolic tumor burden with different thresholds have no substantial difference from each other; thus, the current clinical standard with a fixed absolute threshold value of SUV > 4.0 is a feasible option compared with other methods with fixed relative or background threshold values. In contrast to certain recommendations such as thresholding with an SUV > 2.5, thresholding methods that recommend SUV values < 4.0 should rather be avoided to enable easier applicability in automated software segmentation by avoiding manual modification to exclude organs or structures with physiologic uptake [18], [25], [26]. However, given the limitations of our study such as retrospective design and limited cohort size, this study should be regarded as a preliminary investigation, and further research with larger, prospective cohorts is warranted to validate our results.

## Ethics Approval

All procedures were carried out in accordance with the ethical standards of the institutional and/or national research committees and with the 1964 Helsinki declaration and its later amendments or comparable ethical standards. The study received approval (study number: 2023–2618) from the Ethical Committee of Medical Faculty of Heinrich-Heine-University Duesseldorf, Germany.

## Funding statement

The authors declare that no funds, grants, or other support was received during the preparation of this manuscript.

## CRediT authorship contribution statement

**Eduards Mamlins:** Writing – review & editing, Visualization, Supervision. **Emil Novruzov:** Writing – original draft. **Helena Anne Peters:** Data curation. **Kai Jannusch:** Methodology. **Guido Kobbe:** Validation. **Gerald Antoch:** Methodology. **Frederik L. Giesel:** Conceptualization, Supervision. **Christina Antke:** Validation. **Ben-Niklas Baermann:** Conceptualization. **Jutta Rox:** Resources. **Sascha Dietrich:** Resources. **Johannes C. Fischer:** Resources.

## Declaration of Competing Interest

The authors declare the following financial interests/personal relationships which may be considered as potential competing interests: BNB received travel support from Kite Gilead and Medac and speaker honoraria from Incyte. He has membership at GLA and EBMT and an advisory role at Kite Gilead. GK received honoraria from MSD, Pfizer, Amgen, Novartis, Gilead, BMSCelgene, Abbvie, Biotest, Takeda, Eurocept, Jazz, Medac, and Eurocept. He received lecture fees from MSD, Pfizer, Amgen, Novartis, Gilead, BMSCelgene, Abbvie, Biotest, Takeda, Eurocept, and Jazz. The other authors declare no conflicts of interest regarding this manuscript. If there are other authors, they declare that they have no known competing financial interests or personal relationships that could have appeared to influence the work reported in this paper.

## Data Availability

The data used and/or analyzed during the current study are available from the corresponding author upon reasonable request.
