# Supplementary material for: The predictive power of baseline metabolic and volumetric [18F]FDG PET parameters with different thresholds for early therapy failure and mortality risk in DLBCL patients undergoing CAR-T-cell therapy
Source: Eur J Radiol Open. 2024 Dec 17;14:100619. doi: 10.1016/j.ejro.2024.100619 (PMC11719856; doi:10.1016/j.ejro.2024.100619)
Supplement: Supplementary file 1 — Supplementary material [file mmc1.docx]

**Supplemental Table (S1)**

| Site | University Hospital Düsseldorf | |
| --- | --- | --- |
| PET/CT scanner | Biograph mCT 128, Siemens | |
| Modality | Low dose CT | Full dose (ce)CT |
| CT reference (mAs) | 40 | 190 |
| CT peak kilovoltage (kV) | 120 | 120 |
| CT slice thickness (mm) | 1,5 | 1,5 |
| CT slice increment (mm) | 1 | 1 |
| PET reconstruction | OSEM algorithm | OSEM algorithm |
| Iterations | 4 | 4 |
| Subsets | 8 | 8 |
| Matrix | 200 x 200 | 200 x 200 |
| Corrections | Gaussian FWHM 2.0 mm | Gaussian FWHM 2.0 mm |

**Figure 1:** Graphical illustration of metabolic and volumetric PET parameters at baseline with respect to early therapy response **a)** metabolic parameters **b)** volumetric parameters.

**Figure 2:** An overview of the comparison of ROC curves of metabolic (**a**) and volumetric (**b**) PET parameters for the cohort with early therapy failure.

**Figure 3:** Graphical illustration of metabolic and volumetric PET parameters at baseline with respect to mortality.

**Figure 4:** Overview of the comparison of ROC curves of metabolic and volumetric PET parameters with respect to mortality risk (a, b).

**Figure 5:** Exemplary automated segmentation of the baseline tumor burden with 4 different thresholding methods in a 67-year-old female patient with abdominally relapsed DLBCL: **A)** fixed absolute threshold of SUV_max_ > 4.0, **B)** fixed relative threshold of >40% **C)** background threshold with a liver SUV value + 2 SDs, **D)** background threshold with a liver SUV value.
